# A Markov model of fibrosis development in nonalcoholic fatty liver disease predicts fibrosis progression in clinical cohorts

**DOI:** 10.1002/psp4.13052

**Published:** 2023-10-03

**Authors:** Jane Knöchel, Linnéa Bergenholm, Eman Ibrahim, Stergios Kechagias, Sara Hansson, Mathias Liljeblad, Patrik Nasr, Björn Carlsson, Mattias Ekstedt, Sebastian Ueckert

**Affiliations:** ^1^ Clinical Pharmacology and Quantitative Pharmacology Clinical Pharmacology & Safety Sciences, R&D, AstraZeneca Gothenburg Sweden; ^2^ DMPK, Research and Early Development, Cardiovascular, Renal and Metabolism (CVRM), BioPharmaceuticals R&D, AstraZeneca Gothenburg Sweden; ^3^ Department of Pharmacy Uppsala University Uppsala Sweden; ^4^ Department of Health, Medicine, and Caring Sciences Linköping University Linköping Sweden; ^5^ Translational Science and Experimental Medicine, Research and Early Development Cardiovascular, Renal and Metabolism (CVRM), BioPharmaceuticals R&D, AstraZeneca Gothenburg Sweden

## Abstract

Disease progression in nonalcoholic steatohepatitis (NASH) is highly heterogenous and remains poorly understood. Fibrosis stage is currently the best predictor for development of end‐stage liver disease and mortality. Better understanding and quantifying the impact of factors affecting NASH and fibrosis is essential to inform a clinical study design. We developed a population Markov model to describe the transition probability between fibrosis stages and mortality using a unique clinical nonalcoholic fatty liver disease cohort with serial biopsies over 3 decades. We evaluated covariate effects on all model parameters and performed clinical trial simulations to predict the fibrosis progression rate for external clinical cohorts. All parameters were estimated with good precision. Age and diagnosis of type 2 diabetes (T2D) were found to be significant predictors in the model. Increase in hepatic steatosis between visits was the most important predictor for progression of fibrosis. Fibrosis progression rate (FPR) was twofold higher for fibrosis stages 0 and 1 (F0‐1) compared to fibrosis stage 2 and 3 (F2‐3). A twofold increase in FPR was observed for T2D. A two‐point steatosis worsening increased the FPR 11‐fold. Predicted fibrosis progression was in good agreement with data from external clinical cohorts. Our fibrosis progression model shows that patient selection, particularly initial fibrosis stage distribution, can significantly impact fibrosis progression and as such the window for assessing drug efficacy in clinical trials. Our work highlights the increase in hepatic steatosis as the most important factor in increasing FPR, emphasizing the importance of well‐defined lifestyle advise for reducing variability in NASH progression during clinical trials.

**Study Highlights**

**WHAT IS THE CURRENT KNOWLEDGE ON THE TOPIC?**

The best predictor for liver‐related morbidity and mortality in non‐alcoholic fatty liver disease (NAFLD) is fibrosis. Fibrosis progression is challenging to characterize due to the slow progressive nature of the disease with high heterogeneity and sampling limitations.

**WHAT QUESTION DID THIS STUDY ADDRESS?**

What is the rate of fibrosis progression in NAFLD and how is it impacted by patient characteristics and/or other factors?

**WHAT DOES THIS STUDY ADD TO OUR KNOWLEDGE?**

This study utilizes a unique clinical data source that allows evaluation of longitudinal changes in fibrosis. The developed population fibrosis Markov model includes the impact of age, type 2 diabetes, and hepatic steatosis on the fibrosis progression. Fibrosis progression predicted by the model achieved an overall good agreement with an external clinical cohort.

**HOW MIGHT THIS CHANGE DRUG DISCOVERY, DEVELOPMENT, AND/OR THERAPEUTICS?**

The population fibrosis Markov model can be a useful tool for clinical trial design in nonalcoholic steatohepatitis indication.

## INTRODUCTION

Nonalcoholic steatohepatitis (NASH), the more severe form of nonalcoholic fatty liver disease (NAFLD), is a major cause of liver‐related morbidity and mortality worldwide with no approved pharmacological treatment options.^1^ NAFLD is characterized by steatosis, defined as excess accumulation of fat in the liver (>5%). In case of inflammation and cell injury (ballooning) setting in, the patient has transitioned to NASH. NASH is formally established by histopathological assessment (performing a liver biopsy).^2^ The chronic inflammation can further lead to fibrosis, a scarring of the tissue characterized by the accumulation of extra cellular matrix proteins, such as collagen. Currently, fibrosis stage is the best predictor for the development of end‐stage liver disease (also called cirrhosis, characterized by all healthy tissue being replaced by scar tissue in the liver), liver‐related mortality, and all‐cause mortality.^3^, ^4^, ^5^ Therefore, understanding fibrosis progression and regression is important for the identification of high‐risk patients, design, and enrollment in clinical trials of disease‐modifying pharmacological agents and tailoring of life‐style interventions for these patients.

Previous meta‐analyses have shown that people with NASH progress faster to advanced fibrosis stages than people with NAFLD.^6^, ^7^, ^8^ In Singh et al. and Le et al., the results suggested that fibrosis progression could be dependent on fibrosis stage at baseline.^7^, ^8^ However, the impact of patients' characteristics on fibrosis progression and regression could not be assessed fully, due to lack of access to patient‐level data and the chosen approach. A quantitative, model‐based approach to accurately predict the future development of fibrosis and to identify factors that impact fibrosis progression on an individual patient level is still missing.

Fibrosis stage is assessed histologically by liver biopsy.^2^ NAFLD with no fibrosis is classified as fibrosis stage 0. Stage 4 represent cirrhotic disease and is subdivided into compensated and decompensated cirrhosis, as people presenting stages up to decompensated cirrhosis are asymptomatic or have only minor symptoms. For mathematical purposes, fibrosis stage data needs to be treated as an ordered categorical variable, as the development of the next stage depends on presentation of the previous stage.

Markov models describe the probability for an individual to be in a particular state by modeling the probability of the initial state, as well as the transition probabilities between states.^9^ In contrast to the proportional odds model, Markov models incorporate dependence between consecutive observations and allow to assess how covariates affect each transition rate individually.^9^ In this work, fibrosis stage data were available from a unique longitudinal clinical cohort following people up to 3 decades with varying time intervals at which disease status was assessed.^10^, ^11^ To account for this, the best model option is the continuous‐time Markov model.

Although the mixed‐effect continuous‐time Markov modeling approach is very established for other types of ordered categorical data, such as improvement score in rheumatoid arthritis,^12^, ^13^ fatigue, hand‐and‐foot syndrome,^14^ and Likert pain score,^9^ it has not yet been applied to the fibrosis stage in NAFLD. Developing models for a disease like NAFLD is challenging for several reasons. First, repeat biopsies are vital to identify factors contributing to fibrosis progression rate. However, biopsies are invasive procedures and, therefore, have restrictions on how frequently they can be performed. Consequently, cohorts with sequential biopsy data are rare. Moreover, the design of clinical trials to detect changes in fibrosis is challenging due to the high variability originating from slow disease progression^7^ with high heterogenity^15^ and sampling limitations.^16^, ^17^ However, a general Markov modeling approach has been used to predict the economic and clinical burden of NAFLD using a series of interlinked Markov chains.^18^

In this work, we aimed to quantify differences in fibrosis progression based on fibrosis stage at baseline, patient‐specific factors, as well as levels of circulating biomarkers by building a population continuous‐time Markov model.

## METHODS

### Cohort description

We used a Swedish longitudinal NAFLD cohort consisting of 129 well‐characterized participants with biopsy‐proven NAFLD (Table S1). It is part of a prospective cohort study with biochemical, clinical, and histological data collected at up to three visits spanning over a total of up to 3 decades.^10^, ^11^ Further details on patient consent and sample collection can be found in Text S1. Liver fibrosis stage is assessed using the Brunt scoring system.^2^ People with NAFLD presenting no fibrosis in the liver biopsy were classified as having stage 0. Besides the fibrosis staging, the histological assessment of the liver biopsy includes scoring steatosis, hepatocellular ballooning, and lobular inflammation. The NAFLD activity score (NAS), which ranges from 0 to 8, is an unweighted sum of these scores. The time interval to the first follow‐up is 13.8 (SD = 1.5) years and another 10.3 (SD = 3.9) years to the second follow‐up (Table S1). In total, 58 people died during the entire study period and their causes of death were collected by reviewing the medical records and information from the Registry of Causes of Death. Furthermore, 17 patients were hospitalized due to development of decompensated cirrhosis during the entire duration of the study. As indicated in Table S1, there were 20 people at follow‐up 1 and 26 people at follow‐up 2 who refused to undergo another liver biopsy but still agreed to the biochemical blood assessment.

### Software and data analysis

The software package NONMEM, version 7.3.0 (Icon Development Solutions) was used for model development. Model fitting was performed in a Linux environment (CentOS 7) with GFortran FORTRAN Compiler, version 5.2 (Gnu Compiler Collection). Nonmem2R version 0.2.1 (CRAN.R‐project.org/package=nonmem2R), PsN, version 4.4.8. (psn.sourceforge.net) and R, version 3.5.1 (R‐project, www.r‐project.org) was used for the exploratory analysis, executing NONMEM runs and post‐processing of NONMEM output, for example, to assess goodness‐of‐fit. Mrgsolve version 0.8.12 (CRAN.R‐project.org/package=mrgsolve) was used to perform clinical trial simulations.

### Fibrosis progression model

**FIGURE 1:**
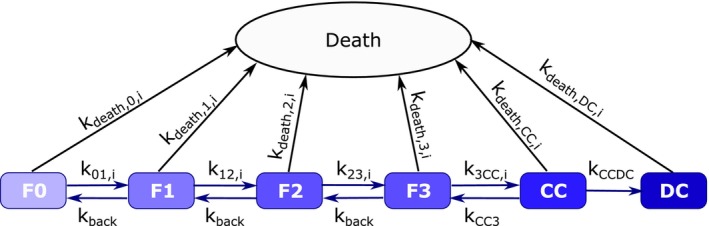
Schematic representation of continuous Markov model for fibrosis progression in nonalcoholic fatty liver disease/nonalcoholic steatohepatitis. Forward transition rates are denoted *k*_*xy*, (*i*)_ giving the transition from stage *x* to stage *y* with subscript *i* added in case that covariates affect transition rate. Backward transition rates are denoted *k*_back_.F0, fibrosis stage 0; F1, fibrosis stage 1; F2, fibrosis stage 2; F3, fibrosis stage 3; CC, compensated cirrhosis; DC, decompensated cirrhosis.

The continuous‐time Markov model was developed using a total of seven compartments: (1) fibrosis stage 0, (2) fibrosis stage 1, (3) fibrosis stage 2, (4) fibrosis stage 3, (5) compensated cirrhosis, (6) de‐compensated cirrhosis, and (7) death (Figure 1). For compartments 1 to 6, transition rates were assumed to occur only between neighboring compartments (e.g., no “jumping”). Back transition rates were included between all, except for compartments 6 (i.e., decompensated cirrhosis) and 7 (i.e., death). The model was initialized by the first observation for each patient.

The data analysis included an investigation of the potential influence of demographic covariates (such as age, body mass index [BMI], type 2 diabetes [T2D], and sex), histological covariates (such as NAS, steatosis, hepatocellular ballooning, and lobular inflammation) and plasma level covariates (such as alanine aminotransferase and aspartate aminotransferase) on fibrosis progression. The effect of BMI was tested both as a continuous and categorical covariate by defining two BMI groups (e.g., dichotomizing the patients into obese and non‐obese by a cutoff of 30 kg/m^2^). The same approach was applied to age, where the cutoff was set at 55 years based on previous findings of more severe steatohepatitis in patients above 55 years.^19^

Covariates were included in the model based on (i) their statistical significance, (ii) uncertainty in model parameters, (iii) diagnostic plots, and (iv) clinical relevance. Both a step‐wise covariate modeling analysis and a manual stepwise approach was performed using forward inclusion (for df = 1, *α* = 0.05) and backward elimination (for df = 1, *α* = 0.01). Each covariate effect was tested on each transition rate separately and in combination (e.g., on all forward rates). Continuous covariates were also incorporated in five different ways, as (i) baseline covariate value, (ii) change from baseline covariate value, (iii) combination of (i) and (ii), (iv) change from previous visit, and (v) standard covariate^20^ (Equation 1). A special case was age, here, instead of the usual step‐wise constant approximation by NONMEM, we continuously updated age based on the integration time.

(1) $$P_{\mathrm{pop}}=\theta_{p}∙\left(1+\theta_{\mathrm{COV}}∙\left(\mathrm{COV}-\mathrm{CO}V_{\text{median}}\right)\right)$$

Furthermore, both time‐varying covariate approaches (next observation carried backward and last observation carried forward) as well as linear interpolation were tested. Additional information around the covariate modeling is given in Tables S3 and S4, as well as Text S2.

The final Markov model was validated with several approaches. First, model evaluations based on relative standard errors and visual predictive checks (VPCs) were performed. Stratification (e.g., based on covariates) was used when appropriate to ensure that the model performs adequately across important subgroups of the data.

### Model simulation and application

We performed clinical trial simulations to compare the model predicted fibrosis progression rate (FPR) with the FPR calculated in a meta‐analysis as well as the mean fibrosis stage from the placebo arm response of seven clinical trials at the end of the study.

For each set of clinical trial simulations, we designed clinical cohorts to mimic the patient characteristics age, T2D, steatosis (change from baseline) and baseline fibrosis stage distribution, as well as number of patients in the placebo arm that was given in the publications. For each study, 1000 cohorts were simulated with baseline characteristics sampled from a normal distribution with the reported mean and SD for age and according to the reported proportions for T2D, steatosis, and fibrosis. Baseline characteristics and longitudinal information were reported for all studies expect for longitudinal information of steatosis for two out of the 15 clinical cohorts. For these two cohorts, we assumed no change in steatosis (i.e., removing the impact of steatosis on fibrosis progression). The virtual cohorts were then simulated over each individual study length.

In the first clinical trial simulation, we compared our model predictions to the results of a previous published meata‐analysis^7^ of fibrosis progression. This meta‐analysis was performed using individual data acquired from 11 published NAFLD cohorts with at least two biopsies. Overall average the FPR was assessed by averaging individually calculated progression rates (e.g., difference in fibrosis stage between the first and last biopsy divided by the time between biopsies in years). The individual FPR was calculated for all patients in each of the 1000 simulated cohorts, and then summarized as mean FPR. In the calculation of the FPR, compensated cirrhosis (CC) and decompensated cirrhosis (DC) were both considered as fibrosis stage 4 and death was not taken into account.

In the second clinical trial simulation, we compared our model predictions against the placebo arm response in seven clinical trials (NCT00492700,^21^ NCT00590161,^22^ NCT01237119,^23^ NCT02006498,^24^ N0192119052,^25^ NCT00323414,^26^ and NCT02970942^27^). Clinical trials were included in this analysis if they fulfilled the following criteria: placebo‐controlled trial of patients with biopsy proven NAFLD/NASH, where baseline fibrosis stage distribution was reported, and patients were evaluated by a second liver biopsy at the end of treatment, details on the patient characteristics in these trials can be found in Table S5. We compared the distribution of the 1000 simulated mean fibrosis stage to the observed mean fibrosis stage in the placebo arm of each trial, and, when available, to the proportion of patients in each fibrosis stage at the end of the study.

Finally, we performed simulations to demonstrate the value of the developed model for assessing (i) the effect of patient characteristics on fibrosis progression rate and (ii) the effect of baseline distribution of fibrosis stage on fibrosis progression, mimicking the behavior of a placebo arm in a clinical trial. For the latter assessment, we defined four cohorts where the average baseline fibrosis stage was 2, whereas the distribution differed, for example, equal distribution across stages 0–4 or half the patients in stage 1 and half in stage 3. In case of 40 patients per trial, this gives eight patients in stage 0–4 each or 20 patients in stages 1 and 3, respectively. All the other patient characteristics were assumed to be the same between the different simulated trials. The patient and baseline characteristics assumed for these clinical trial simulations are summarized in Table S6.

## RESULTS

### Fibrosis progression model and factors associated with fibrosis progression

The final continuous‐time Markov model is given in Figure 1. Forward transition rates were estimated to be different for all transitions. Backward transition rates ($k_{\text{back}}$) were estimated to be the same for stages 1–3, whereas it was fixed to a small value for CC ($k_{\mathrm{CC}3}$), as this transition was not observed in the data. At DC the backward transition was assumed to be negligible, and once patients reach this stage there is acute deterioration in liver function and risk for life‐threatening complications.^28^ Probability of death was assumed to increase exponentially with increasing fibrosis stage (Equation 2) in line with previously published findings of all‐cause mortality increasing with fibrosis stage.^29^, ^30^

Significant covariates for the final model were age, T2D, and change in steatosis grade (DSTEA). The complete list of tested covariates can be found in Table S3. The forward transition rates increased exponentially with steatosis (Equations (3), (4), (5), (6)) and increased by an estimated factor for T2D. The parameters from the fibrosis progression Markov model are given in Table 1. The transition rates increase with increasing fibrosis stage. The forward transition rate from CC to DC is around two times higher compared to that between the lower fibrosis stages (0–3).

**TABLE 1:** Parameter estimates for the fibrosis progression Markov model.

Parameter	Estimate (RSE)	Bootstrap estimate (90% CI)
$k_{01}$ (1/year)	0.066 (0.27)	0.063 (0.04–0.1)
$k_{12}$ (1/year)	0.076 (0.31)	0.075 (0.046–0.12)
$k_{23}$ (1/year)	0.078 (0.29)	0.076 (0.044–0.13)
$k_{3\mathrm{CC}}$ (1/year)	0.11 (0.35)	0.11 (0.056–0.19)
$k_{\text{back}}$ (1/year)	0.061 (0.29)	0.06 (0.035–0.096)
$k_{\text{CCDC}}$ (1/year)	0.15 (0.40)	0.15 (0.075–0.36)
$k_{\text{death}}$ (1/year)	0.071 (0.17)	0.07 (0.045–0.11)
$\lambda_{\text{death}}$	0.21 (0.32)	0.21 (0.049–0.4)
$k_{\mathrm{age}}$	0.12 (0.14)	0.12 (0.094–0.15)
$k_{\text{DSTEA}}$	1.8 (0.37)	1.9 (0.92–3.5)
$\beta_{T2D}$	0.36 (0.26)	0.38 (0.21–0.58)

*Note*: Bootstrap estimate and CI were based on 2000 samples. All estimates were rounded to 3 significant digits.

Abbreviations: CI, confidence interval; RSE, relative standard error.

VPCs using 1000 simulated data sets were made to evaluate the predictive ability of the fibrosis progression Markov model. The model predictions agreed with the observed data (see Figure S1) as well as the frequency of observed transitions between fibrosis stages (see Figure S2).

The death transition rate for fibrosis stage *x* is calculated as:

(2) $$k_{\text{death},x,i}=k_{\text{death}}∙\exp\left(\lambda_{\text{death}}∙x\right)∙kf_{\mathrm{age}}$$

The equation for *kf* describing the impact of DSTEA and T2D on the forward transition rates is given below:

(3) $$k_{01,i}=k_{01}∙\exp\left(k_{\text{DSTEA}}∙\text{DSTEA}\right)∙k_{T2D}$$

(4) $$k_{12,i}=k_{12}∙\exp\left(k_{\text{DSTEA}}∙\text{DSTEA}\right)∙k_{T2D}$$

(5) $$k_{23,i}=k_{23}∙\exp\left(k_{\text{DSTEA}}∙\text{DSTEA}\right)∙k_{T2D}$$

(6) $$k_{3\mathrm{CC},i}=k_{3\mathrm{CC}}∙\exp\left(k_{\text{DSTEA}}∙\text{DSTEA}\right)∙k_{T2D}$$

with $k_{T2D}$ given as

(7) $$k_{T2D}=1+\beta_{T2D}$$

where $\beta_{T2D}$ equal to zero for people without T2D.

The impact of age on the death rate is given by the following equation:

(8) $$kf_{\mathrm{age}}=\exp\left(k_{\mathrm{age}}∙\left(\mathrm{Age}-76.9\right)\right)$$

### Model simulation and application

#### Model application – predicting previously published meta‐analysis results

**FIGURE 2:**
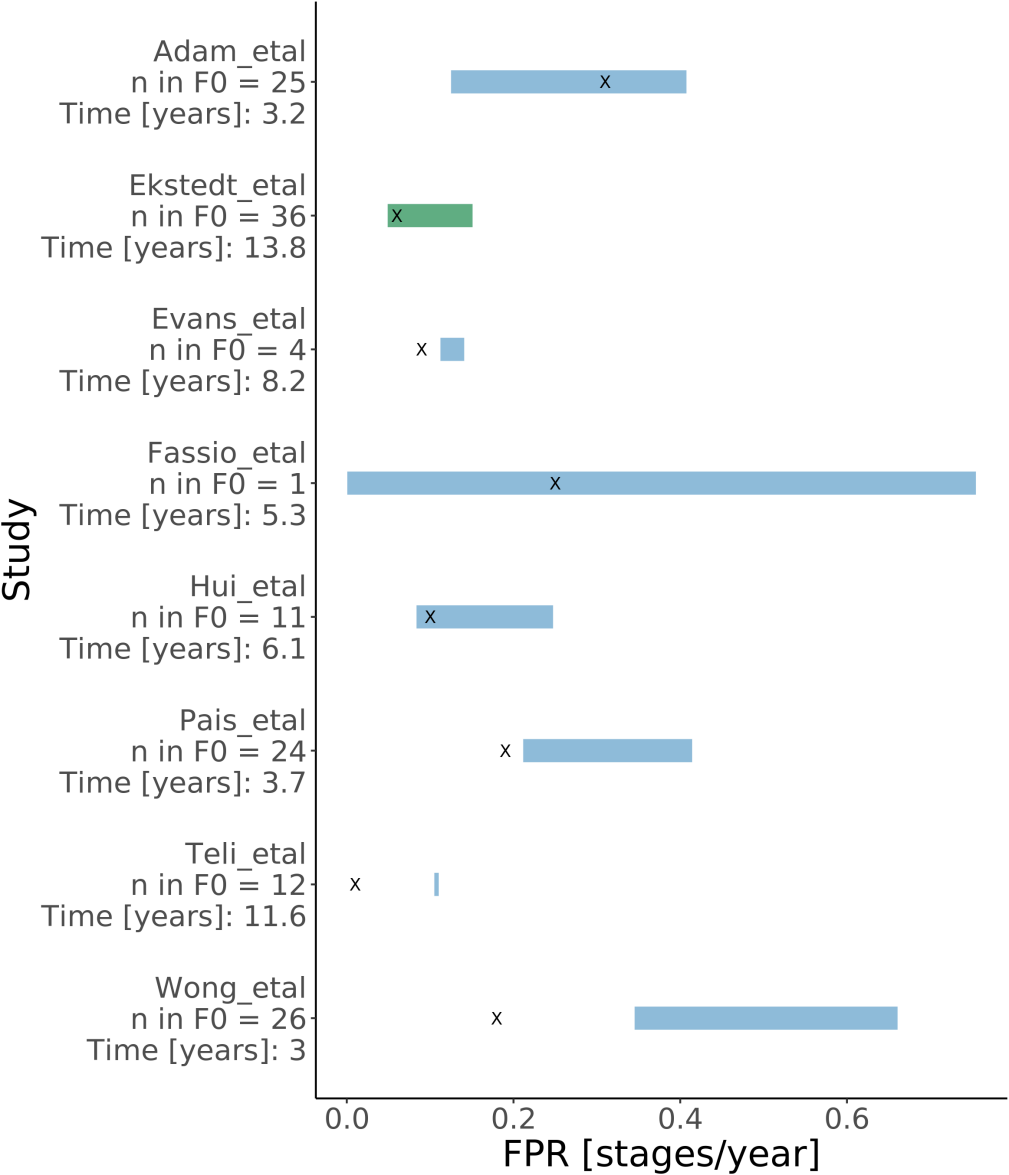
Comparison of the predicted FPR by the Markov model (shared blue box), the analysis cohort Ekstedt et al. is highlighted in green, and calculated FPR for fibrosis stage 0 using meta‐analysis of eight clinical nonalcoholic fatty liver disease cohorts with paired biopsies (*x*). FPR, fibrosis progression rate.

In a previous meta‐analysis,^7^ the average FPR was assessed for 11 NAFLD cohorts by averaging individually calculated progression rates for each study. We performed simulations of hypothetical cohorts mimicking the cohorts included in this meta‐analysis. The median and 95 percentiles for the predicted FPR based on the 1000 simulated cohorts was compared to the results of the meta‐analysis (Figure 2). The Markov model predicted the FPR for Adam et al., Ekstedt et al. (our analysis cohort), Fassio et al., and Hui et al. very well. For Evans et al. and Pais et al., the model predictions deviated slightly from the observed fibrosis progression rate, for Teli et al. and Wong et al., the model predictions are more than two times higher than the observed FPR.

#### Model application – predicting clinical trial outcome for placebo

We performed clinical trial simulation with the continuous‐time Markov model of fibrosis in a similar manner, as was proposed in the work by Chan et al.^31^ to predict the observed outcomes in the placebo arms of seven clinical trials (NCT00492700,^21^ NCT00590161,^22^ NCT01237119,^23^ NCT02006498,^24^ N0192119052,^25^ NCT00323414,^26^ and NCT02970942^27^). Simulations of 1000 sampled cohorts mimicking each trial cohort were generated and the predicted progression data for each simulated patient was calculated and averaged to achieve summary statistics for each clinical trial. As the initial distribution of fibrosis stages is very different among the seven trials, the temporal predictions were exhibiting different dynamics with respect to the progression in each stage (Figure S2).

**FIGURE 3:**
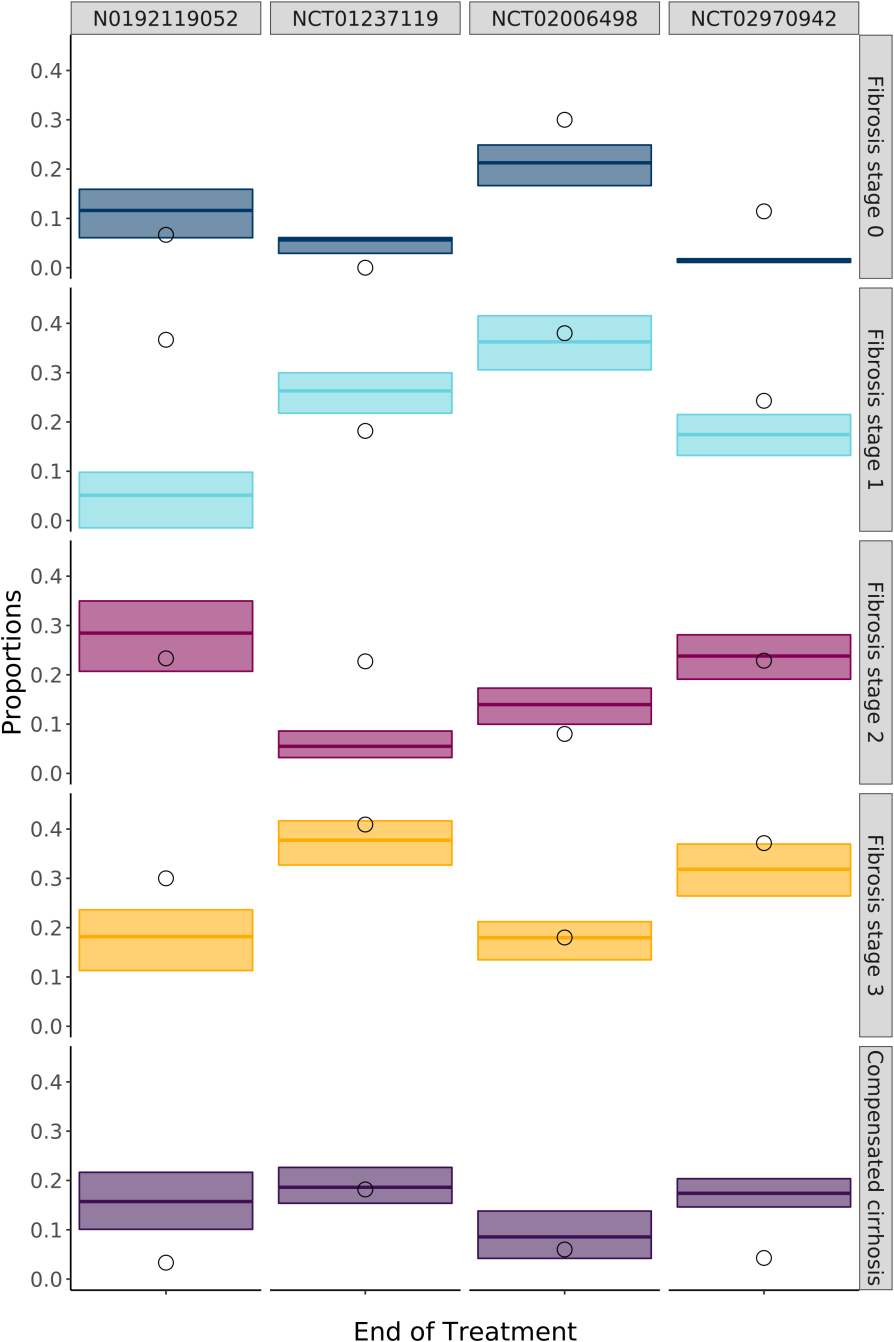
Model prediction of fibrosis stage for placebo arms overlayed with observations at end of study for NCT01237119, N0192119052, NCT02006498, and NCT02970942. Given are the observed mean fibrosis stage at end of treatment (EOT) of placebo arm from four clinical trials^23^, ^24^, ^25^, ^27^ shown as open circles as well as the predicted median (5th and 95th quantile) shown as boxplots of simulated means of fibrosis stage at EOT of 1000 clinical trial simulations.

Comparing the observed and predicted mean of fibrosis stage in Table 2, it became evident that the model captured the fibrosis progression independent of increase or decrease for N0192119052, NCT00323414, NCT00590161, and NCT1237119 very accurately. For the remaining three trials, the model predicted a larger increase in fibrosis, based on the fibrosis mean observed at the end of the study, than was seen in the data. Looking at the proportions of patients at each stage for clinical study NCT01237119, it showed that whereas the overall mean was well‐predicted, there was a clear underprediction of proportion of patients in fibrosis stage 2 (Figure 3). When the proportions of patients in each fibrosis stages for the other studies are compared with the observed means, the model generally performed well. The model underpredicted the proportion of patients with fibrosis stage 1 for N0192119052 but generally captured the proportion of patients with this fibrosis stage well for the other studies.

**TABLE 2:** Summary of observed and predicted fibrosis for placebo arm of seven clinical trials.

Clinical trial ID	Fibrosis at baseline mean	Fibrosis at EOT mean	Predicted fibrosis at EOT median (2.5th and 97.5th quantiles)
N0192119052	2.10	2.07	1.95 (1.62, 2.25)
NCT00323414	1.94	2.00	2.02 (1.89, 2.26)
NCT00492700	2.29	2.11	2.51 (2.38, 2.69)
NCT00590161	1.72	2.12	2.05 (1.87, 2.29)
NCT01237119	2.41	2.50	2.46 (2.38, 2.57)
NCT02006498	1.22	1.32	1.59 (1.42, 1.78)
NCT02970942	2.17	1.99	2.53 (2.43, 2.65)

*Note*: Given are the observed mean fibrosis stage at baseline and EOT of the placebo arm from seven clinical trials^21^, ^22^, ^23^, ^24^, ^25^, ^26^, ^27^ as well as the predicted median (5th and 95th quantiles) of simulated means of fibrosis stage at EOT of 1000 clinical trial simulations.

Abbreviation: EOT, end of treatment.

#### Model simulation – impact of patient characteristics on FPR

**FIGURE 4:**
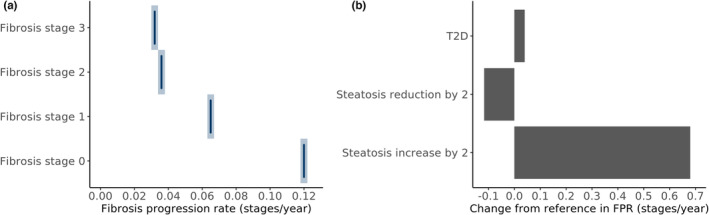
Impact of baseline fibrosis stage (a) and change in steatosis or T2D (b) on FPRs. Reference is defined as fibrosis stage 0 at baseline, with 50 years of age, no diabetes, steatosis grade 2, and no change in steatosis observed. FPR, fibrosis progression rate; T2D, type 2 diabetes mellitus.

In this section, we assessed the impact of change in steatosis from −2 to +2, age 50 to 60 years, and patients with or without T2D on the FPR. To do this, we have performed clinical trial simulations of hypothetical cohorts of patient with a fixed set of covariates. A visualization of the impact of fibrosis stage at baseline and the impact of steatosis and T2D on FPR for patients starting at fibrosis stage 0 is given in Figure 4a,b. In general, the FPR was reduced as the participants progress through the fibrosis stages, with the highest FPR seen for fibrosis stage 0 with FPR of 0.12 stages/year (Figure 4a). The progression rate from fibrosis stage 0 increased by 0.68 stages/year if steatosis was increased by two grades during the same period or decreased by 0.117 stages/year resulting in an FPR of 0.003 stages/year if steatosis decreased by two grades (Figure 4b). The impact of T2D was less pronounced in comparison to steatosis and resulted in an increase of FPR by 0.04 stages/year resulting in a FPR of 0.16 stages/year. This shows that the selected patient characteristics had a large impact on the observed rate of fibrosis progression. A full table assessing all possible combinations can be found in Table S6. In summary, FPR was 1.3 up to 1.8 times faster for patients with T2D without any change in steatosis. An increase in steatosis by two grades resulted in 6.2 up to 11‐fold faster progression, with the maximal increase in progression seen for people with fibrosis stage 2 at baseline. A reduction in steatosis resulted in 1.6 up to 40‐fold slower FPR, with the slowest FPR seen for fibrosis stage 0 at baseline where a decrease of two steatosis grades results in NAFLD resolution.

#### Model simulation – impact of fibrosis stage distribution at baseline

We simulated data for four hypothetical placebo arms, to study the impact of baseline fibrosis stage distribution on the end of treatment assessment of fibrosis. The proportion of T2D and steatosis grades were kept identical, whereas four different sets of fibrosis stage distributions at baseline were used, giving a mean fibrosis stage 2 in a total of 40 patients. The model simulations are given in Figure S3 and fibrosis stage is summarized in Table 3. In the simulated time span of a year, the mean fibrosis stage was comparable for all studies in contrast to the FPR which was highest for studies 1 and 4. Interestingly, studies 1 and 2 resulted in a highly similar mean prediction of fibrosis stage for up to 5 years, despite different FPR of 0.0665 and 0.0629. The predicted FPR for study 1 was 1.2 times higher than for study 3 (20% faster). The lowest FPR after 5 years was observed in study 3.

**TABLE 3:** Summary of predicted fibrosis stage mean for four hypothetical clinical trials with different baseline fibrosis distribution.

Study	Number of patients per fibrosis stage at baseline					Baseline fibrosis stage mean	Predicted fibrosis stage mean / FPR		
							After 1 year	After 2 years	After 5 year
	0	1	2	3	4				
1	10	10	0	10	10	2	2.04/0.0746	2.06/0.0723	2.08/0.0665
2	8	8	8	8	8	2	2.03/0.0689	2.06/0.0674	2.08/0.0629
3	0	10	20	10	0	2	2.03/0.0625	2.04/0.0615	2.06/0.0579
4	0	20	0	20	0	2	2.03/0.0702	2.05/0.0682	2.05/0.0628

Abbreviation: FPR, fibrosis progression rate.

## DISCUSSION

The prevalence of NAFLD is increasing worldwide and already now surpasses all other common liver diseases, as such, the clinical focus is to identify people at risk of developing liver‐related complications.^32^ The best predictor for liver‐related morbidity and mortality in NAFLD is the stage of fibrosis, which is challenging to characterize due to high variability originating from slow disease progression^7^ with high heterogenity^15^ and sampling limitations.^16^, ^17^ Herein, we present a continuous population Markov model to quantify the fibrosis progression in patients with NAFLD. The model is the first of its kind for fibrosis progression and is based on a unique longitudinal NAFLD cohort with repeat biopsies spanning up to 3 decades.

We found that the rate of fibrosis progression increases with increasing fibrosis stages in patients with NAFLD. The highest transition rate was observed for CC to DC.^33^ Stepwise covariate modeling identified the presence of T2D and change in biopsy assessed steatosis as statistically significant covariates of the forward rates for stages from F0 to CC. T2D is a well‐known risk factor for NAFLD/NASH^34^ and has been previously identified as a predictor for fibrosis progression^15^ and mortality.^35^ We found that fibrosis progression in the early stages was up to two times faster in patients with T2D compared to people without diabetes. Obesity, defined as BMI greater than or equal to 30, is an established risk factor for NAFLD^34^ and highly correlated with liver steatosis.^36^ In our model, change in steatosis had a profound effect on fibrosis progression for early fibrosis stages, where a worsening of two points for steatosis led to 6.2 up to 11‐fold faster progression, whereas an improvement by two points led to a similar degree of fibrosis rate reduction from 1.6 up to 40‐fold. The maximal impact of a progression of two grades in steatosis on FPR was seen for baseline fibrosis stage 2. For the regression of two grades in steatosis, the largest impact was seen at baseline fibrosis stage 0, which resulted in an FPR that was close to 0 (40‐fold lower), as, in this case, NAFLD resolution was observed. To put this into context of NAS, which includes steatosis score, a two‐point improvement in NAS is typically considered a meaningful clinical outcome.^37^ In addition to age, sex, race, and ethnicity are considered risk factors for NAFLD.^32^ However, sex was not found to be a significant covariate in this analysis. The potential impact of ethnicity or race could not be evaluated in this analysis due to the homogeneity of the cohort population.

Our analysis spotlights the importance of the initial fibrosis distribution on the observed fibrosis progression after 1–5 years. Our Markov model derived that the inclusion of a majority of individuals with fibrosis stage 2, a common mean in clinical trials, resulted in the slowest progression of the placebo arm in the hypothetical clinical trial. In the case of recruitment of 50% of people with fibrosis stage 2, the progression rate was the lowest. Overall, these results demonstrated the importance of initial fibrosis stage distribution for the fibrosis progression observed in clinical trials for NAFLD. However, current noninvasive scores, such as FIB4, NFS,^38^ and ADAPT,^39^ can thus far only be used to identify advanced fibrosis stage (fibrosis stage higher than 2). Therefore, noninvasive scores or biomarkers will prove invaluable to include the patient population of interest based on baseline fibrosis stage distribution.

Previous meta‐analysis presented diverging results with respect to FPR ranging from 0.03 ± 0.53 stages/year^6^ to 0.13 (95% confidence interval: 0.07–0.18) stages/year for patients with baseline stage F0.^7^ In this analysis, we demonstrate by using the summary characteristics of the cohorts investigated by Singh et al.,^7^ that both the proportions of patients in each fibrosis stage as well as in each steatosis grade are important predictors of FPR and need to be accounted for when calculating the FPR. The Markov model predictions were able to capture the results for each cohort well except for Teli et al. and Wong et al. As NASH is a fast‐evolving field, it could be speculated that Teli et al. were using different techniques to assess fibrosis stage and classify the disease in the 1980s. The cohort of Wong et al. stands out from all the remaining cohorts with a high steatosis progression, the Markov model consequently predicts a higher fibrosis progression for this cohort. It could be speculated that there are some underlying racial differences that were not captured by the model. The meta‐analysis by Singh et al. successfully calculated FPR for baseline fibrosis stages F0 and F1,^7^ the Markov model can extend this analysis to provide FPR for each baseline fibrosis stage (Table S1). We would like to note that whereas the usage of FPR has been well‐established in the field, the higher FPR for lower stages is a mere reflection of the fact that fibrosis stage caps at 4 and thus starting from higher stages signified less room for worsening of fibrosis.

In the present analysis, neither liver heterogeneity nor any misspecification in fibrosis stage were considered. Due to the long‐time interval between successive visits in the analysis cohort, it was impossible to differentiate progression or regression from inherent variability or random error. Although all participants were invited to perform repeat biopsies independent of their disease status, not all agreed to it, potentially introducing a selection bias into the model based on the patient characteristics. No obvious difference in disease severity could be identified (data not shown). Because of the limited number of data points per individual, interindividual variability could not be identified. Currently, the Markov model includes a link between change in hepatic steatosis grade and fibrosis progression, and therefore requires the knowledge of steatosis grade to predict fibrosis. Whereas, in this analysis, steatosis was assessed via liver biopsy, in recent years, imaging techniques to assess liver fat content have become more common, such as magnetic resonance imaging proton density fat fraction (MRI‐PDFF). A relative MRI‐PDFF decline of 30% has been shown to predict fibrosis regression.^40^ By using the link between MRI‐PDFF and steatosis, one could build on the model in the future to predict histology outcome without the performance of a liver biopsy. Important additional future extensions of this work may be the separation of liver‐related risk and cardiovascular mortality from other‐cause mortality and investigation of the specific risks for hepatocellular carcinoma and liver transplantation. The Markov model provides a useful framework for incorporating novel biomarkers as predictors for fibrosis progression and continued development of the model will improve its generalizability and reliability. Including longitudinal cohorts, with shorter time between follow‐up, will provide assurance that time periods less than 1 or 2 years are accurately captured in the model as well. The comparison of fibrosis progression predicted by the Markov model with cohorts included in the meta‐analysis by Singh et al. and seven clinical placebo arms showed overall a very good agreement and no general over‐ or underprediction of specific fibrosis stages could be identified.

The strengths of this analysis were that the fibrosis transition rates could be reliably estimated based on a unique longitudinal cohort and the resulting population Markov model could be used to predict the fibrosis progression of specific patient cohorts accounting for the baseline distribution of fibrosis stages and considering the impact of T2D, steatosis, and age. The presented model will prove useful in the future to account for the placebo effect in upcoming therapeutic trials for NASH, as it allows prediction of fibrosis progression for the placebo arm based on the chosen inclusion and exclusion criteria for the trial, informing already ahead of time of potential fibrosis progression or regression.

## CONCLUSIONS

We present a population Markov model for fibrosis progression in patients with NAFLD and show that patient selection in particular initial fibrosis stage distribution can have clear impact on fibrosis progression and as such the window for assessing drug efficacy in clinical trials.

## AUTHOR CONTRIBUTIONS

All authors wrote the manuscript. J.K., L.B., E.I., and S.U. designed the research. J.K. and E.I. performed the research and analyzed the data.

## FUNDING INFORMATION

No funding was received for this work.

## CONFLICT OF INTEREST STATEMENT

J.K., L.B., S.H., M.J., B.C., and S.U. were employed by AstraZeneca and may own stock. M.E. was on the advisory board for AMRA Medical AB, his department received research funds from AstraZeneca and lecture fees from Mediplast. All other authors declared no competing interests for this work. As an Editor‐in‐Training for *CPT: Pharmacometrics & Systems Pharmacology*, Jane Knöchel was not involved in the review or decision process for this paper.

## Supporting information


Appendix S1
