# Supplementary material for: A Markov model of fibrosis development in nonalcoholic fatty liver disease predicts fibrosis progression in clinical cohorts
Source: CPT Pharmacometrics Syst Pharmacol. 2023 Oct 3;12(12):2038–49. doi: 10.1002/psp4.13052 (PMC10725269; doi:10.1002/psp4.13052)
Supplement: Supplementary file 1 — Appendix S1 [file PSP4-12-2038-s001.pdf]

Supplement material for A Markov model of fibrosis development in patients with Non-Alcoholic Fatty Liver Disease predicts fibrosis progression in clinical cohorts

Jane Knöchel[1], Linnéa Bergenholm[2], Eman Ibrahim[3], Stergios Kechagias[4], Sara Hansson[5], Mathias Liljeblad[5], Patrik Nasr[4], Björn Carlsson[6], Mattias Ekstedt[4], Sebastian Ueckert[1]

1. Clinical Pharmacology and Quantitative Pharmacology, Clinical Pharmacology & Safety Sciences, R&D, AstraZeneca, Gothenburg, Sweden
2. DMPK, Research and Early Development, Cardiovascular, Renal and Metabolism (CVRM), BioPharmaceuticals R&D, AstraZeneca, Gothenburg, Sweden
3. Pharmacometrics, Department of Pharmacy, Uppsala University
4. Department of Health, Medicine, and Caring Sciences, Linköping University, Linköping, Sweden
5. Translational Science and Experimental Medicine, Research and Early Development, Cardiovascular, Renal and Metabolism (CVRM), BioPharmaceuticals R&D, AstraZeneca, Gothenburg, Sweden
6. Early Clinical Development, Research and Early Development, Cardiovascular, Renal and Metabolism (CVRM), BioPharmaceuticals R&D, AstraZeneca, Gothenburg, Sweden

* Correspondence to jane.knochel@astrazeneca.com

Supplement Text S1: Sample collection and informed consent

Written informed consent was obtained from each patient included in the study and the study protocol conforms to the ethical guidelines of the 1975 Declaration of Helsinki as reflected in a priori approval by the institution's human research committee.

Human biopsy samples were collected after an over-night fast. Liver steatosis grade, lobular inflammation grade, hepatocellular ballooning grade and the fibrosis stage were scored in the HE- and PRS-stained liver sections using a conventional histopathological method [2,23].

Supplement Table S 1 Demographics, clinical and biochemical characteristics of longitudinal NAFLD cohort

Variable	Baseline	1st follow up	2nd follow up
Number of participants	129	88	59
Time between two consecutive visits [years]		13.8 (1.5)	10.3 (3.9)
Age [years]	51.0 (12.9)	61.0 (11.0)	66.5 (10.3)
Women	42 (32.6)	26 (29.5)	11 (18.6)
T2D	14 (10.9)	51 (58.0)	39 (66.1)
BMI [kg/m^2]	28.3 (3.8)	29.1 (4.7)	29.1 (4.0)
ALT [IU/L]	76.2 (43.3)	59.9 (35.0)	50.0 (34.4)
AST [IU/L]	44.7 (22.9)	35.0 (15.1)	41.7 (23.0)
ALP [IU/L]	60.6 (32.7)	64.7 (37.4)	72.1 (31.1)
GGT [ukat/L]	1.6 (2.0)	1.3 (1.1)	1.6 (1.6)
Number of patients with liver biopsy	129	68	33
Change in steatosis		-0.4 (1.1)	-0.4 (1.1)
Steatosis			
0	0 (0.0)	3 (4.4)	6 (18.2)
1	36 (27.9)	28 (41.2)	9 (27.3)
2	25 (19.4)	16 (23.5)	15 (45.5)
3	68 (52.7)	21 (30.9)	3 (9.1)
Fibrosis stage			
0	60 (46.5)	24 (35.3)	14 (42.4)
1	31 (24.0)	22 (32.4)	5 (15.2)
2	22 (17.1)	11 (16.2)	10 (30.3)
3	12 (9.3)	7 (10.3)	4 (12.1)
4	4 (3.1)	4 (5.9)	0 (0.0)

Parameters are listed as mean (SD) for continuous variables and as total numbers (%) for categorical variables. T2D: type 2 diabetes, ALT: Alanine Aminotransferase, AST: Aspartate Aminotransferase, ALP: Alkaline Phosphatase, GGT: Gamma-glutamyl Transferase

Supplement Figure S 1 Visual predictive check for fibrosis progression Markov model

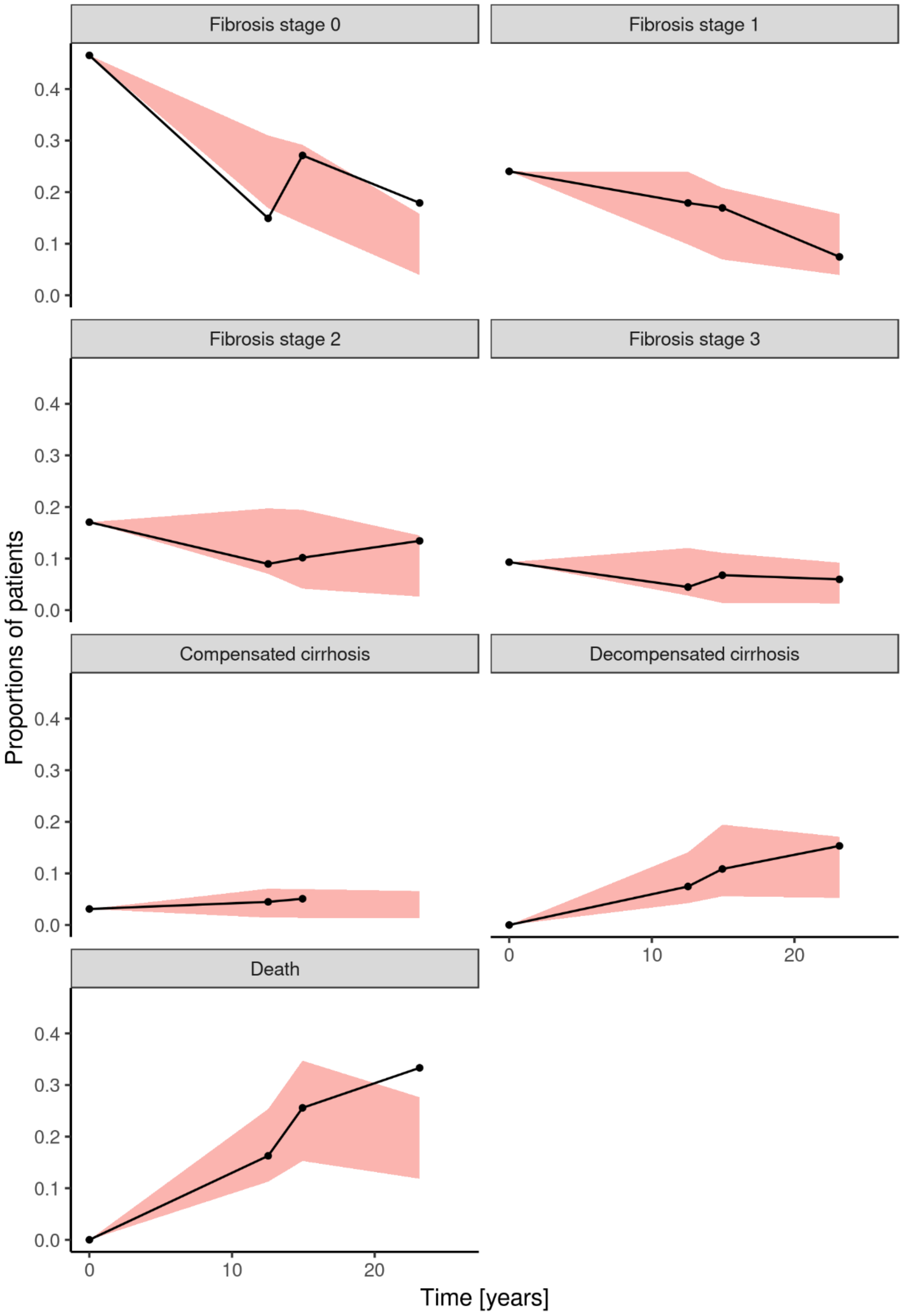

The solid black lines represent the proportions of NAFLD patients at each fibrosis stage or that died over time. The shaded areas are the 95% confidence intervals of the model prediction generated from 1000 simulation.

Supplement Figure S 2 Visual predictive check of transition rates for fibrosis in Markov model
The solid black lines represent the proportions of NAFLD patients at each transition over time. The

shaded areas are the 95% confidence intervals of the model prediction generated from 1000 simulation.

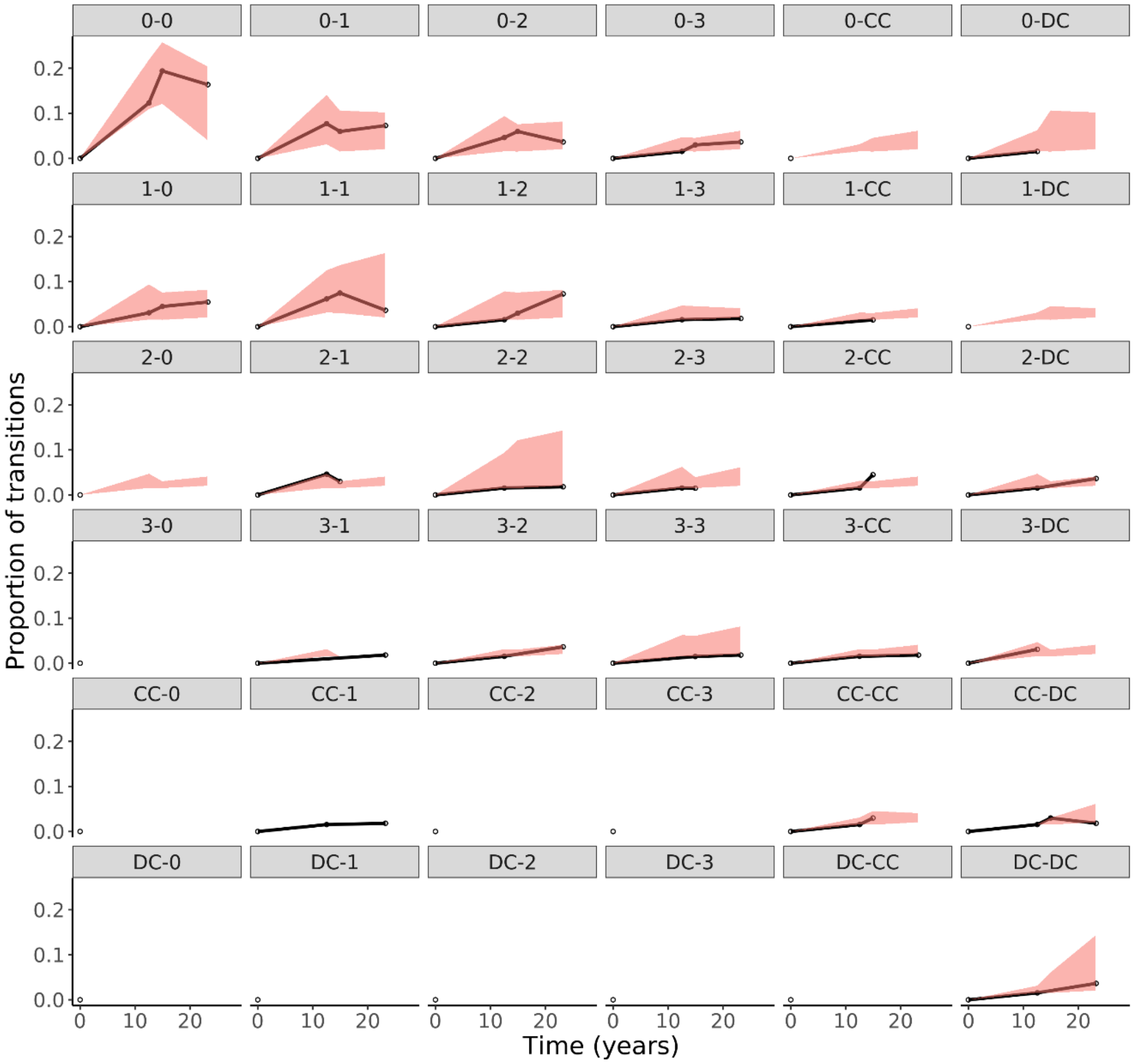

Supplement Table S 2 Predicted fibrosis progression rate (FPR) by the Markov model

Baseline fibrosis stage	Markov model FPR (stages/year) Median (95 percentiles)
0	0.27 (0.19 - 0.35)
1	0.18 (0.11 - 0.26)
2	0.11 (0.063 - 0.17)
3	0.056 (0.025 - 0.086)

FPR: fibrosis progression rate

Supplement Figure S *3* Model prediction of fibrosis stage over time for placebo arms in seven clinical trials. The solid line represents the median of the simulated means and the shaded area represents the 5th and 95th quantile of the simulated means of the 1000 clinical trials simulation with different compositions for the baseline characteristics resulting in the same summary statistics as given in the respective trial publication.

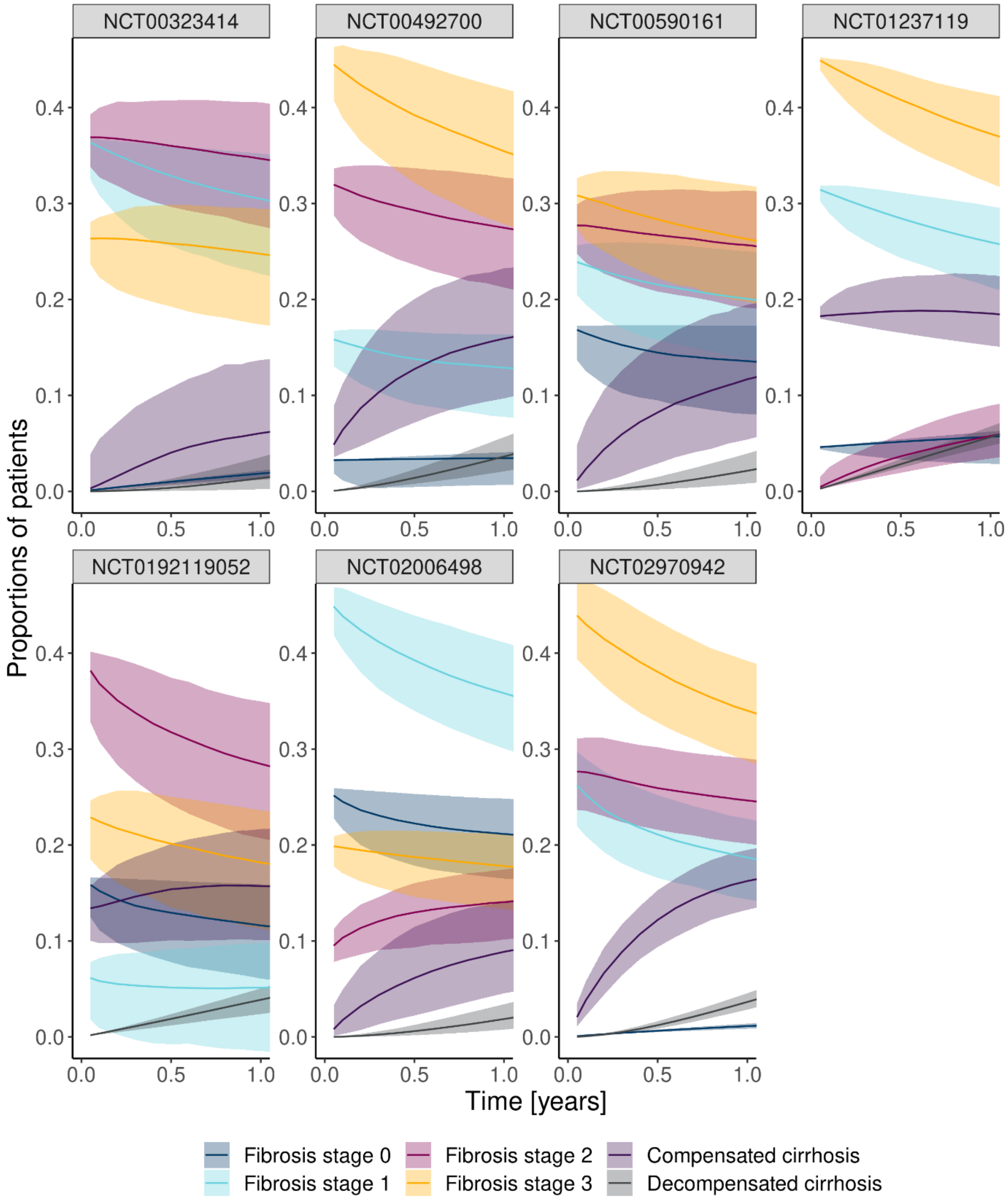

Supplement Figure S 4 Model simulation of fibrosis stage progression over time for four hypothetical clinical trials with different baseline fibrosis stage distribution

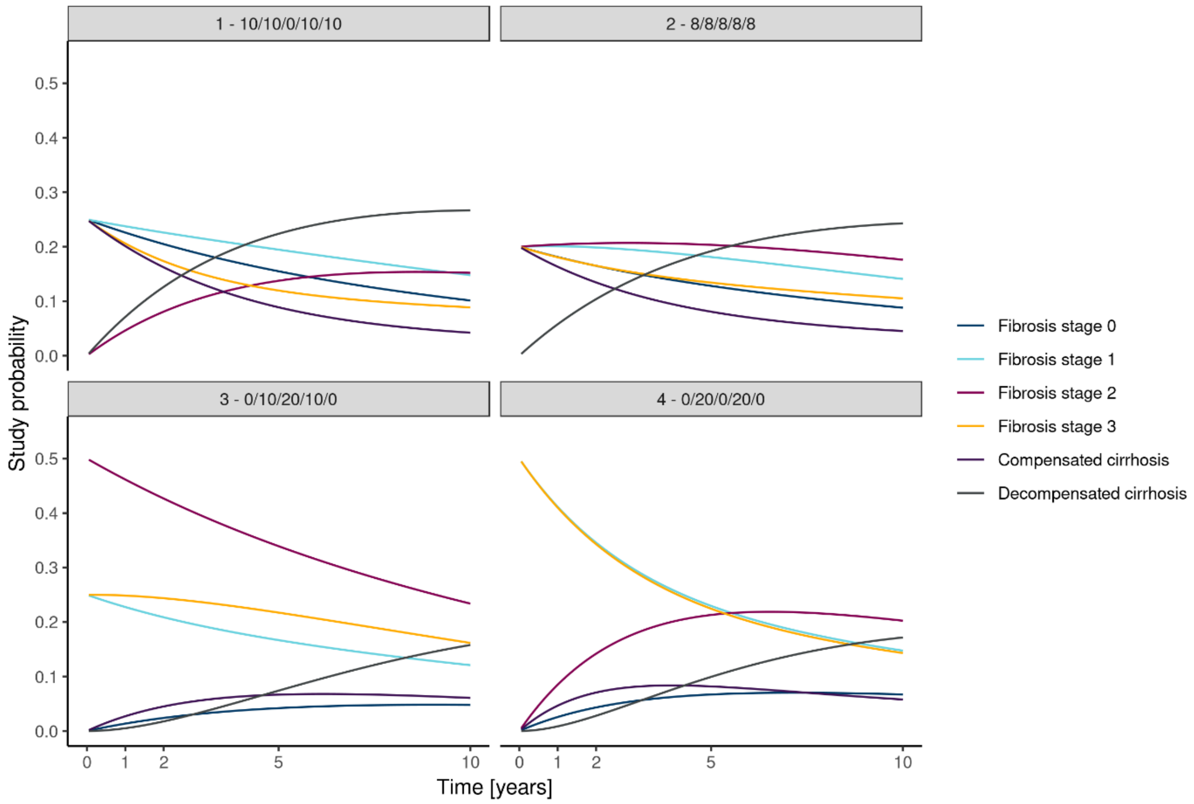

Supplement Text S2: Covariate modelling

The cohort was initiated in 1988 without assessment of all the standard clinical parameters that are collected nowadays. Therefore, parameters not available at baseline were excluded from the covariate model building step. Furthermore, parameter with more than 40% missing values were excluded as well. For the remaining parameters an initial graphical visualisation guided the final potential covariate selection. The final list of potential covariates and on which parameter they were tested on is given in Supplement Table S2. The percentage of missing values for these covariates is given in Supplement Table S3. Depending on the type of the covariate missing values were imputed by (i) median/mean, (ii) last observation carried forward[1] or (iii) a new category. The default for interpolation of time-varying covariates is next observation carried backwards (NOCB) resulting in step functions[2], we, therefore, coded the dataset to allow for last observation carried forward (LOCF) for the interval between two observation.

Due to the larger number of covariates to be tested and the 11 model parameters on which they should be tested on, a multiple step approach was taken:

i. testing all covariates on each parameter
ii. testing all covariates that came out as significant in step i.
iii. testing all covariates that came out as significant in step i only for obese patients

Supplement Table S 3 List of covariates investigated for their potential impact on the Markov model parameters

Covariate	Unit	Parameter	Comment
Age	years	K_death	Age clearly linked to mortality and potentially to faster or slower progression with increasing age
ALT	U/L	K_01,k_12,k_23, k_34,k_45,kf_12, kf_23,kf34,kf123, kf1234,kf234	All these parameter have been previously identified either to correlate to fibrosis progression or to correlate to fibrosis stage.
AST	U/L		
AST/ALT	ratio		
BMI	kg/m^2		
Platelets	10^9/L		
Sex	Categorical variable		
obesity	Categorical variable		
T2D	Categorical variable		
NAS	Categorical variable		
BALLO	Categorical variable		
STEA	Categorical variable		
LOB_INF	Categorical variable		
PORT_INF	Categorical variable		
PERI_INF	Categorical variable		

ALT: Alanine-Aminotransferase; AST: Aspartate transaminase; AST/ALT: ratio of AST and ALT; BMI: Body mass index; T2D: type 2 diabetes; NAS: NAFLD activity score; BALLO: hepatocellular ballooning; STEA: steatosis score; LOB_INF: lobular inflammation; PORT_INF: portal inflammation; PERI_INF: peripheral inflammation. For each covariate impact of baseline, change between visits and variable as standard covariate was considered. Kf_xy represents the joint testing of a covariate on both k_x-1,x and k_y-1,y.

Supplement Table S 4 Summary of percent missing values for the covariates of interest in the NAFLD cohort data

Variable	Percent missing data at baseline [%]	Percent missing data at 1st follow up [%]	Percent missing data at 2nd follow up [%]	Percent missing data overall [%]
Age	0.0	0.0	0.0	0.0
ALT	0.0	2.3	3.4	1.4
AST	0.0	2.3	1.7	1.1
BALLO	0.0	22.7	45.8	17.0
BMI	3.1	0.0	1.7	1.8
LOB_INF	0.0	22.7	47.5	17.4
NAS	0.0	22.7	49.1	17.8
Platelets	12.4	1.1	0.0	6.2

Variable	Percent missing data at baseline [%]	Percent missing data at 1st follow up [%]	Percent missing data at 2nd follow up [%]	Percent missing data overall [%]
ALT/AST	0.0	2.3	3.4	1.4
STEA	0.0	22.7	44.1	16.7
T2D	0.0	0.0	0.0	0.0

ALT: Alanine-Aminotransferase; AST: Aspartate transaminase; AST/ALT: ratio of AST and ALT; BMI: Body mass index; T2D: type 2 diabetes; NAS: NAFLD activity score; BALLO: hepatocellular ballooning; STEA: steatosis score; LOB_INF: lobular inflammation;

Supplement Table S 5 Summary of clinical trial placebo arm baseline and EOT characteristics used for clinical trial simulations.

Variable	N0192119052[3]	NCT00323414[4]	NCT00492700[5]	NCT00590161[6]	NCT01237119[7]	NCT02006498[8]	NCT02970942[9]
Number of patients	30	19	31	29	26	50	80
Trial duration [weeks]	48	48	52	52	48	48	72
Age	51.7 (35.5)	49.8 (12.1)	54.1 (10.4)	49.6 (9.6)	52 (12)	50.1 (10.2)	52.4 (10.8)
BMI	30.8 (4.1)	35.7 (7)	30.5 (4.4)	34 (5.4)	37.7 (6.2)	31 (4.6)	36.1 (6.6)
Steatosis	2.20	2.06	1.97	2.14	1.90	2.10	2.00
Fibrosis	2.10	1.94	2.29	1.72	2.41	1.22	2.17
T2D	0	19	11	4	8	30	50
Fibrosis stage 0	5	0	1	5	1*	13	0
Fibrosis stage 1	2	7	5	7	7*	23	22
Fibrosis stage 2	12	7	10	8	0*	4	22
Fibrosis stage 3	7	5	14	9	10*	10	36
Fibrosis stage 4**	4	0	1	0	4*	0	0
Change in steatosis	0.03	-0.59	-0.10	-0.04	-0.40	-0.20	-0.16
Steatosis at EOT	2.23	1.47	1.87	2.10	1.50	1.90	1.84

* Endpoint was only available for 22 patients, thus only used 22 patients with original reported patient characteristics in the simulation. ** Fibrosis stage 4 was interpreted as compensated cirrhosis. Continuous variables are given as mean (SD) and categorical variables as absolute numbers of patients. Steatosis and fibrosis was reported as mean of number of patients per score. BMI: Body mass index; T2D: type 2 diabetes; EOT, End of treatment

Supplement Table S 6 Summary of assumed patient and baseline characteristics in the clinical trials simulation to assess impact of fibrosis stage distribution at baseline.

**Variable**	**1**	**2**	**3**	**4**
Number of patients	40	40	40	40
Trial duration [weeks]	52	52	52	52
Age	52.35 (10.4)	52.35 (10.4)	52.35 (10.4)	52.35 (10.4)
BMI	30.85 (6.1)	30.85 (6.1)	30.85 (6.1)	30.85 (6.1)
Steatosis	2.23	2.23	2.23	2.23
T2D	26.7	26.7	26.7	26.7
Fibrosis stage 0	10	8	0	0
Fibrosis stage 1	10	8	10	20
Fibrosis stage 2	0	8	20	0
Fibrosis stage 3	10	8	10	20
Fibrosis stage 4	10	8	0	0
Change in steatosis	0	0	0	0

BMI: Body mass index; T2D: type 2 diabetes;

Supplement Table S 7 Impact of patient characteristics on fibrosis proression rate for different baseline fibrosis stages.

T2D<br><br>(0=No, 1=Yes)	Baseline fibrosis stage	Change in steatosis	FPR<br><br>(stages/year)	Relative FPR with reference based on fibrosis baseline, no change in steatosis and w/o T2D
0	0	-2	0.003	0.025
0	0	0	0.120	1.000
0	0	2	0.800	6.700
1	0	-2	0.004	0.034
1	0	0	0.160	1.300
1	0	2	0.800	6.700
0	1	-2	-0.050	-0.770
0	1	0	0.065	1.000
0	1	2	0.600	9.200
1	1	-2	-0.049	-0.750
1	1	0	0.100	1.500
1	1	2	0.600	9.200
0	2	-2	-0.054	-1.500
0	2	0	0.036	1.000
0	2	2	0.400	11.000
1	2	-2	-0.053	-1.500
1	2	0	0.068	1.900
1	2	2	0.400	11.000
0	3	-2	-0.057	-1.800
0	3	0	0.032	1.000
0	3	2	0.200	6.200
1	3	-2	-0.056	-1.800
1	3	0	0.056	1.800
1	3	2	0.200	6.200

T2D: type 2 diabetes; FPR: fibrosis progression rate

Supplement Text S3: NONMEM model code for simulation

```
;; 1. Based on: 2066
;; 2. Description: ordered categorical mixed effect model for
fibrosis stage
;; x1. Author: Jane Knoechel
;; 4. Date: 14.01.2023
;; 5. Version: 1
;; 6. Label:
;; Basic model
;; 7. Structural model:
;; transition model/Marcov model CTMM
;; different forward and same backward transfer rates
;; CIRRHOSIS presents as stage 4
;; decompensated CIRRHOSIS presents as stage 5
;; from stage 0 to 1 and vice versa
;; from stage 1 to 2 and vice versa
;; from stage 2 to 3 and vice versa
;; DEATH
;; 8. Covariate model:
;; NONE
;; 9. Inter-individual variability:
;; NO
;; 10. Inter-occasion variability:
;; No
;; 11. Residual variability:
;; none
;; 12. Estimation:
;; COND LAPLACE LIKE NUMERICAL SLOW
$PROBLEM    Transition prop between different fibrosis stages
$INPUT      ID TIME EVID DV CMT AMT AGE BAGE T2D DSTEA IDV
$DATA
../DerivedData/Linkoeping_NONMEM_data_markovModel_14012023_sim.csv
            IGNORE=@ IGNORE(ID.EQ.420)
$SUBROUTINE ADVAN13 TOL=7
$MODEL      COMP=(FIB_0) ;0
            COMP=(FIB_1) ;1
            COMP=(FIB_2) ;2
            COMP=(FIB_3) ;3
            COMP=(FIB_4) ;4
            COMP=(Dec_cir) ;5
            COMP=(DEATH) ;6
$PK
REP=IREP
; Set the probability amount in the rigth compartment  at TIME=0
IF(NEWIND.NE.2) THEN
PDVA=0
PDVB=0
ENDIF

IF(EVID.EQ.0) THEN
PDVB=IDV
ENDIF


IF(PDVB.EQ.0.AND.TIME.EQ.0) THEN
F1=1
```

```
F2=0
F3=0
F4=0
F5=0
ENDIF

IF(PDVB.EQ.1.AND.TIME.EQ.0) THEN
F1=0
F2=1
F3=0
F4=0
F5=0
ENDIF

IF(PDVB.EQ.2.AND.TIME.EQ.0) THEN
F1=0
F2=0
F3=1
F4=0
F5=0
ENDIF

IF(PDVB.EQ.3.AND.TIME.EQ.0) THEN
F1=0
F2=0
F3=0
F4=1
F5=0
ENDIF

IF(PDVB.EQ.4.AND.TIME.EQ.0) THEN
F1=0
F2=0
F3=0
F4=0
F5=1
ENDIF



;------ Turn on/off the correct CPT according to simulated DV-------
-------
; NOTE: the dataset contains 7 rows with AMT=1 and CMT=1 to 7,
respectively
; This allows to turn on the CMT corresponding to the simulated DV
; and to turn off all other CMT
IF(PDVA.EQ.0.AND.TIME.GT.0) THEN
F1=1
F2=0
F3=0
F4=0
F5=0
F6=0
F7=0
ENDIF
IF(PDVA.EQ.1.AND.TIME.GT.0) THEN
```

```
F1=0
F2=1
F3=0
F4=0
F5=0
F6=0
F7=0
ENDIF
IF(PDVA.EQ.2.AND.TIME.GT.0) THEN
F1=0
F2=0
F3=1
F4=0
F5=0
F6=0
F7=0
ENDIF
IF(PDVA.EQ.3.AND.TIME.GT.0) THEN
F1=0
F2=0
F3=0
F4=1
F5=0
F6=0
F7=0
ENDIF
IF(PDVA.EQ.4.AND.TIME.GT.0) THEN
F1=0
F2=0
F3=0
F4=0
F5=1
F6=0
F7=0
ENDIF
IF(PDVA.EQ.5.AND.TIME.GT.0) THEN
F1=0
F2=0
F3=0
F4=0
F5=0
F6=1
F7=0
ENDIF
IF(PDVA.EQ.6.AND.TIME.GT.0) THEN
F1=0
F2=0
F3=0
F4=0
F5=0
F6=0
F7=1
ENDIF

$PK
```

```
;;; KT2D-DEFINITION START
IF(T2D.EQ.0) KT2D = 1  ; Most common
IF(T2D.EQ.1) KT2D = ( 1 + THETA(12))
;;; KT2D-DEFINITION END

;;; KF1234DSTEA-DEFINITION START
KF1234DSTEA = EXP(THETA(13)*DSTEA)
;;; KF1234DSTEA-DEFINITION END

; FIXED-EFFECTS

TVK01 = THETA(1)
TVK12 = THETA(2)
TVK23 = THETA(3)
TVK34 = THETA(4)

TVK10 = THETA(5)
TVK21 = THETA(5)
TVK32 = THETA(5)
TVK43 = THETA(5)

TVK45 = THETA(6)
TVKX  = THETA(7)
TVKL  = THETA(8)
TVKAGE = THETA(9)

TVKF1234  = THETA(10)
TVKF1234 = KF1234DSTEA*TVKF1234

TVK = THETA(11)
TVK = KT2D*TVK


; DEFINE IIV
ZK01 = ETA(1)


; create variables in case of normal event
IF(NEWIND.LE.1) THEN
Stmp = 1
A1 = 1
A2 = 1
A3 = 1
A4 = 1
A5 = 1
A6 = 1
ENDIF

; DEFINE KF1234
KF1234 = TVKF1234

; DEFINE K
K = TVK

; DEFINE KAGE
KAGE = TVKAGE
```

```
; DEFINE K01
K01 = TVK01 *K*KF1234

; DEFINE K12
K12 = TVK12 *K*KF1234


; DEFINE K23
K23 = TVK23*K*KF1234

; DEFINE K34
K34 = TVK34*K*KF1234

; DEFINE K10
K10 = TVK10

; DEFINE K21
K21 = TVK21

; DEFINE K32
K32 = TVK32

; DEFINE K43
K43 = TVK43

; DEFINE K45
K45 = TVK45*K

; DEFINE KXD0
KXD0   = TVKX*EXP( TVKL *0)  ; Death rate  from stages 0/1/2/3/4/5
All-cause mortality
; DEFINE KXD1
KXD1   = TVKX*EXP( TVKL *1)
; DEFINE KXD2
KXD2   = TVKX*EXP( TVKL *2)
; DEFINE KXD3
KXD3   = TVKX*EXP( TVKL *3)
; DEFINE KXD4
KXD4   = TVKX*EXP( TVKL *4)
; DEFINE KXD5
KXD5   = TVKX*EXP( TVKL *5)

;-----------------------------------------------------------------
------------

$DES

CAGE = BAGE+T

KDAGE1 = (EXP(KAGE*(CAGE - 76.9))) ;updated median age for dead

KXD0A = KXD0 * KDAGE1
KXD1A = KXD1 * KDAGE1
KXD2A = KXD2 * KDAGE1
```

```
KXD3A = KXD3 * KDAGE1
KXD4A = KXD4 * KDAGE1
KXD5A = KXD5 * KDAGE1


K01A   = K01
K12A   = K12
K23A   = K23
K34A   = K34
K45A   = K45


; fibrosis stage 0-3
DADT(1) = -K01A * A(1) + K10 * A(2) - KXD0A * A(1)
DADT(2) = -K10 * A(2) + K01A * A(1) - K12A * A(2) + K21 * A(3) -
KXD1A * A(2)
DADT(3) = -K21 * A(3) + K12A * A(2) - K23A * A(3) + K32 * A(4) -
KXD2A * A(3)
DADT(4) = -K32 * A(4) + K23A * A(3) - K34A * A(4) + K43 * A(5) -
KXD3A * A(4)

;fibrosis stage 4 and 5
DADT(5) = -K43 * A(5) + K34A * A(4) - K45A * A(5)-KXD4A * A(5)
DADT(6) =  K45A * A(5) - KXD5A * A(6)

; death compartment
DADT(7) =  KXD0A * A(1)+KXD1A * A(2)+KXD2A * A(3)+KXD3A * A(4)+KXD4A
* A(5)+ KXD5A * A(6)

$ERROR
S  = 1

P_1_tmp = A(1)  ; Probability of observing Stage 0
P_2_tmp = A(2)  ; Probability of observing Stage 1
P_3_tmp = A(3)  ; Probability of observing Stage 2
P_4_tmp = A(4)  ; Probability of observing Stage 3
P_5_tmp = A(5)  ; Probability of observing Stage 4
P_6_tmp = A(6)  ; Probability of observing decompensted cirrhosis
P_7_tmp = A(7)  ; Probability of observing Death

sum_P = P_1_tmp + P_2_tmp + P_3_tmp + P_4_tmp + P_5_tmp + P_6_tmp +
P_7_tmp

; Calculate accumulated probabilities to compute in which cmt
pateints is
P1=P_1_tmp
P2=P1+P_2_tmp
P3=P2+P_3_tmp
P4=P3+P_4_tmp
P5=P4+P_5_tmp
P6=P5+P_6_tmp
P7=1



;------- simulation block --------------------------------------
```

```
IF(ICALL.EQ.4) THEN
CALL RANDOM (2,R)
RAN=R
;--- Initialize internal variables ---
IF(NEWIND.NE.2) THEN
DV2=0
PDVA=0
ENDIF
;--- Simulate fibrosis stage/DEATH/ ---
IF(EVID.EQ.0) THEN
IF(R.LE.P1) DV2=0
IF(R.GT.P1.AND.R.LE.P2) DV2=1
IF(R.GT.P2.AND.R.LE.P3) DV2=2
IF(R.GT.P3.AND.R.LE.P4) DV2=3
IF(R.GT.P4.AND.R.LE.P5) DV2=4
IF(R.GT.P5.AND.R.LE.P6) DV2=5
IF(R.GT.P6.AND.R.LE.P7) DV2=6
ENDIF

IF (TIME.EQ.0) DV2=PDVB


ENDIF ; end of simulation block

;-------- Track the previous simulated stage------------------------
--------
IF(EVID.EQ.0) THEN

PDVA=DV2

ENDIF
Y=DV2

; -----------------Rate Constants-----------------------------------
-------------
$THETA  0.066 FIX ; 1 K01
$THETA  0.076 FIX ; 2 K12
$THETA  0.078 FIX ; 3 K23
$THETA  0.11 FIX ; 4 K34
$THETA  0.061 FIX ; 5 K10 = K21 = K32 = K43
$THETA  0.15 FIX ; 6 K45
$THETA  0.071 FIX ; 7 Kdeath_012345
$THETA  0.21 FIX ; 8 exponent death
$THETA  0.12 FIX ; 9 AGEonDEATH
$THETA  1 FIX ; 10 KF1234
$THETA  1 FIX ; 11 K all
$THETA  1.8 FIX ; 12 KT2D1 rel
$THETA  0.36 FIX ; 13 KF1234DSTEA lin
$OMEGA  0  FIX  ;          1
;$SIGMA  0  FIX ; 1

$SIMULATION (0238721) (14207 UNIFORM) ONLYSIM NOPREDICTION
$TABLE      ID TIME EVID DV CMT AMT AGE BAGE T2D DSTEA IDV  DV2
sum_P
            FILE=run2072vpc NOPRINT ONEHEADER
```

References

1. Wählby, U., Thomson, A. H., Milligan, P. A. & Karlsson, M. O. Models for time-varying covariates in population pharmacokinetic- pharmacodynamic analysis. *Br J Clin Pharmacol* **58**, 367–377 (2004).

2. Beal, S. L., Sheiner, L. B., Boeckmann, A. & Bauer, R. J. NONMEM users guides. *San Francisco, CA: University of California* (2010).

3. Aithal, G. P. *et al.* Randomized, Placebo-Controlled Trial of Pioglitazone in Nondiabetic Subjects With Nonalcoholic Steatohepatitis. *Gastroenterology* **135**, 1176–1184 (2008).

4. Dasarathy, S. *et al.* Double-blind randomized placebo-controlled clinical trial of omega 3 fatty acids for the treatment of diabetic patients with nonalcoholic steatohepatitis. *J Clin Gastroenterol* **49**, 137–144 (2015).

5. Ratziu, V. *et al.* Rosiglitazone for Nonalcoholic Steatohepatitis: One-Year Results of the Randomized Placebo-Controlled Fatty Liver Improvement With Rosiglitazone Therapy (FLIRT) Trial. *Gastroenterology* **135**, 100–110 (2008).

6. Zein, C. O. *et al.* Pentoxifylline improves nonalcoholic steatohepatitis: A randomized placebo-controlled trial. *Hepatology* **54**, 1610–1619 (2011).

7. Armstrong, M. J. *et al.* Liraglutide safety and efficacy in patients with non-alcoholic steatohepatitis (LEAN): A multicentre, double-blind, randomised, placebo-controlled phase 2 study. *The Lancet* **387**, 679–690 (2016).

8. Wah Kheong, C., Nik Mustapha, N. R. & Mahadeva, S. A Randomized Trial of Silymarin for the Treatment of Nonalcoholic Steatohepatitis. *Clinical Gastroenterology and Hepatology* **15**, 1940-1949.e8 (2017).

9. Newsome, P. N. *et al.* A Placebo-Controlled Trial of Subcutaneous Semaglutide in Nonalcoholic Steatohepatitis. *New England Journal of Medicine* **384**, 1113–1124 (2021).
